# Mother’s lifestyle: development of a questionnaire to assess a determinant of children’s health. A cross-sectional study: Estilo de vida materno: desenvolvimento de um questionário para avaliar um determinante da saúde da criança. Um estudo transversal

**DOI:** 10.1590/1516-3180.2013.7260013

**Published:** 2014-10-28

**Authors:** Erica Bezerra Nobre, Sandra Josefina Ferraz Ellero Grisi, Alexandre Archanjo Ferraro

**Affiliations:** I MSc. Nutritionist, University Restaurant, Fundação Universidade de Brasília (FUB), Brasília, Federal District, Brazil.; II PhD. Full Professor, Department of Pediatrics, Faculdade de Medicina da Universidade de São Paulo (FMUSP), São Paulo, Brazil.; III PhD. Associate Professor, Department of Pediatrics, Faculdade de Medicina da Universidade de São Paulo (FMUSP), São Paulo, Brazil.

**Keywords:** Life style, Child, preschool, Mother-child relations, Questionnaires, Validation studies [publication type]

## Abstract

**CONTEXT AND OBJECTIVE:**

Lifestyle includes the personal attitudes or behavioral patterns that result in risks or benefits to the individual’s own health or that of others. Children’s health is particularly determined by their mother’s lifestyle. The objective here was to develop and evaluate the reliability of a questionnaire capable of describing the lifestyles of preschoolers’ mothers in terms of their activities, interests, opinions and values.

**DESIGN AND SETTING:**

Cross-sectional study conducted in a public university.

**METHODS:**

This study was conducted between January 2010 and March 2011, among 255 mothers of preschoolers living in the southeastern region of the municipality of São Paulo. A proportional stratified random probabilistic sample with two strata was selected: schools were drawn and then the children. Three instruments found in the literature were used to create the lifestyle questionnaire. The questionnaire was developed in eight stages: preliminary pretest, cultural adaptation, second pretest, pilot study, semantic correction and adaptation, third pretest, final research and final retest. Cronbach’s alpha and pairwise correlation coefficients were used.

**RESULTS:**

The Cronbach’s alpha value in the final version was 0.83 and the pre and post-test pairwise correlation coefficients were greater than 0.5. Factor analysis identified five factors that explained 73.51% of the correlation variance. As a result, seven variables were eliminated from the questionnaire.

**CONCLUSIONS:**

The questionnaire described five lifestyle domains, with good reliability, and can be used in combination with preschoolers’ health and nutritional outcomes.

## RESUMO

**CONTEXTO E OBJETIVO:**

Estilo de vida compreende as atitudes pessoais ou padrões de comportamento que trazem riscos ou benefícios para a própria saúde ou de outros. A saúde das crianças é principalmente determinada pelo estilo de vida da mãe. O objetivo foi desenvolver e avaliar a confiabilidade de um questionário capaz de descrever o estilo de vida de mães de pré-escolares em termos de atividades, interesses, opiniões e valores.

**TIPO DE ESTUDO E LOCAL:**

Estudo transversal conduzido em uma universidade pública.

**MÉTODOS:**

Estudo realizado entre janeiro de 2010 e março de 2011 com 255 mães de pré-escolares residentes na Região Sudoeste do Município de São Paulo. Selecionou-se uma amostra probabilística aleatória estratificada proporcional com dois estratos, sendo sorteadas as escolas e depois as crianças. Três instrumentos foram encontrados na literatura e utilizados para compor o questionário de estilo de vida. O desenvolvimento do questionário cumpriu oito etapas: pré-teste preliminar, adaptação cultural, segundo pré-teste, estudo piloto, correção e adaptação semântica, terceiro pré-teste, pesquisa final e reteste. Foram utilizados o alfa de Cronbach e coeficiente de correlação *pairwise*.

**RESULTADOS:**

O valor do alfa de Cronbach na versão final foi de 0,83 e o coeficiente de correlação *pairwise* pré e pós-teste foram maiores que 0,5. A análise fatorial identificou cinco fatores, os quais explicaram 73,51% da variância de correlação. Como resultado, sete variáveis foram eliminadas do questionário.

**CONCLUSÃO:**

O questionário descreveu cinco domínios de estilo de vida, com boa confiabilidade, e pode ser utilizado em associações com aspectos nutricionais e de saúde de pré-escolares.

## INTRODUCTION

For all age groups, including the pediatric age group, diseases associated with behavior and lifestyle are gaining increased attention. Emerging nations are experiencing epidemiological transitions that create new challenges for the healthcare sector. One epidemiological transition is the decrease in pediatric morbidity and mortality due to infectious diseases, which is progressively diverting attention to the healthy development of children who now enjoy longer life expectancies. For adults, this transition has generated new understanding of the genesis of illnesses and has identified the fundamental role of lifestyle. Several studies have assessed the behavioral lifestyles of young adults;^1^^,^^2^^,^^3^^,^^4^^,^^5^^,^^6^^,^^7^^,^^8^ however, little has been done regarding the impact of such lifestyles on children’s health.

Parental lifestyle, especially the mother’s, is a determinant of childhood health because parental lifestyle affects the environment in which the child grows and develops. The effects of this determinant are visible in disease epidemics among children, such as the incidence of overweight or obesity,^9^^,^^10^ and the high rates of mental disorders present in school-age children.^11^

Lifestyle includes personal attitudes or behavioral patterns that result in risks or benefits to personal health or to the health of others.^8^^,^^12^ However, most lifestyle studies concentrate more on behavior and less on the motivation behind these behaviors. This motivation includes attitudes, interests, opinions and values that change an individual’s pattern of life.^13^^,^^14^^,^^15^^,^^16^^,^^17^^,^^18^^,^^19^^,^^20^

In addition to the activities, interests and opinions that are the basis of the mother’s lifestyle, it is necessary to understand the mother’s values. Values are feelings regarding what is important in relation to a person’s goals in life.^21^^,^^22^ Values also explain why people make decisions. Mothers’ personal values need to be understood, because these values translate into particular ways of thinking and acting. Not only the behaviors but also the dimensions involved in the lifestyle of the child’s mother need to be identified.

Currently, no instruments are available in Brazil for describing the mother’s lifestyle, i.e. the activities, interests, opinions and values that are the expression of the person’s pattern of life. Such an instrument could be used in studies relating to preschool child health and nutrition. Children’s behavior, personality and health habits are shaped and values are acquired during the preschool years, and all of these characteristics are obtained primarily through the mother’s influence. The greatest contribution of such an instrument would be to achieve greater capacity to change parental lifestyles through interventions.

## OBJECTIVE

The objective of this project was to develop and evaluate the reliability of a questionnaire capable of describing the lifestyle of preschool-age children’s mothers through their attitudes, interests, opinions and values.

## METHODS

### Population and sample

Between January 2010 and March 2011, a cross-sectional study was conducted among the mothers of preschool-age children (3 to 5 years of age). The target population was the mothers of children who were enrolled in preschools in the Butantã health district in the city of São Paulo (SP, Brazil), which had an estimated population of 427,757 residents in 2010.^23^ The percentages of mothers belonging to each socioeconomic class followed the same distribution as shown by the whole population of the city of São Paulo in 2010. The sampling unit was the mother-child pair.

To select the mothers, a proportional stratified random sample was selected with two strata (“school” and “child”). The sampling grid included the entire list of public and private preschools indexed in the regional school directory of Butantã (59 schools; 37 private and 22 public). The directors of five private schools did not allow their children to participate in the study; in the end, the study included 11 public and 7 private schools.

The criteria for inclusion were that the child needed to be properly enrolled and regularly attending classes of the first and second stage, i.e. the first and second years of preschool. Because of the need to collect each child’s anthropometric data for analysis along with other outcomes, the criterion for exclusion was a neurological deficiency in the child. The type of deficiency for exclusion was not defined. Exclusion was determined after an analysis on the child followed by the mother’s confirmation that the child possessed some type of neurological disorder.

The selection took place in two stages. First, the schools were randomly selected, and the school director was contacted by phone. Then, the children were randomly selected, and an invitation addressed to the mothers was placed in the child’s notebook. Next, the mothers who agreed to participate in the study were contacted by a trained field researcher, by phone. Before the study began, the mother read and signed an informed consent form, to signal agreement to participate in the project.

The sample size was calculated using an α of 0.05, a β of 0.10, and a correlation coefficient of 0.5, thus requiring 38 individuals for the questionnaire to be evaluated.^24^ However, data were collected from 255 mothers in order to analyze outcomes relating to another study on child health and nutrition.

### Study protocol

The interviews were conducted at the mother’s house or at the child’s school without the influence of a third party. For 102 mothers, a re-test was conducted within 15 days.

Data were collected from the mothers regarding age, socioeconomic class and marital status. Marital status was ascertained according to five categories: single, married, separated, divorced or widowed.

Socioeconomic class was evaluated using the Brazilian Economic Classification Criterion of 2007, developed by the Brazilian Association of Market Research Companies (Associação Brasileira de Empresas de Pesquisa; ABEP),^25^ which uses schooling level and the presence of consumer goods in the home to classify individuals into five classes: A, B, C, D and E. Class A has the greatest purchasing power and education level, whereas class E possesses the lowest purchasing power and education level. ABEP’s goal was to obtain a standardized scoring system to economically classify families by estimating their consumption capacity.^25^

### Questionnaire construction

The questionnaire was developed in accordance with Reichenheim and Moraes.^26^ A bibliographic search was conducted to identify projects that evaluated lifestyle questionnaires using the same definition utilized in that study. Three instruments^21^^,^^27^^,^^28^ were found that had already been translated into Portuguese, containing 35 statements regarding values and lifestyle (i.e. personal preferences within various topics), 15 statements regarding self-image (i.e. the image that each person has of their own personality) and 47 statements regarding activities, interests and opinions based on the concept of lifestyle adopted in the present study. However, these instruments had not been validated for the Brazilian population. No Brazilian-validated instrument was found that used the same concepts as those adopted in the present study.

The instrument for the present study was constructed in eight stages: application of the questionnaire statements in a first pretest, cultural adaptation, a second pretest, pilot study, correction and adaptation of the semantics, a third pretest, final study and a re-test. All pre-tests were conducted on a convenience sample of mothers with children in public schools. In this sampling, all the mothers contacted agreed to enter the study, and the tests were conducted either at their home or at the child’s school. The pilot study was conducted in two schools (one public and one private), in the same manner as the pretests. The final study was conducted in the home or in the school environment.

In the first pretest, the three instruments identified were administered to 12 mothers of preschool-age children of different social classes. The following response options were used for the questionnaire on values and lifestyle and for the questionnaire on activities, interests and opinions: “I totally disagree”, “I partially disagree”, “I partially agree” and “I totally agree”. For the self-image questionnaire, the responses followed a Likert scale from 1 to 7.

During instrument development, the usefulness of the items and the ease of understanding by the population were taken into consideration. The need for cultural adaptation and content evaluation was noted, because all the mothers in the first pretest requested explanations for the meaning of certain items. Two of the instruments, i.e. values and lifestyle^21^ and self-image,^27^ for which the psychometric properties were not assessed, were eliminated because they would be difficult for mothers with fewer years of education to complete alone and because the questions were irrelevant within the Brazilian cultural context. Out of the three instruments initially proposed, only 29 selected phrases regarding attitudes, interests and opinions (AIO)^28^ were used. These items were similar to those used by Kucukemiroglu in 1997.^14^ However, the response options on the new form included “I completely disagree”, “I disagree”, “I neither agree nor disagree”, “I agree”, and “I agree completely”. In addition, 24 statements on personal values drafted by an expert in the field were added with the following response options: “not important”, “slightly important”, “important”, “very important”, and “totally important”, since these were the answers that best fitted the type of statement regarding personal values.

The second version of the questionnaire was subjected to a second pretest, which included 10 mothers. This version contained 29 items on activities, interests and opinions and 24 items on personal values. The mothers were asked to indicate whether a question was unclear or whether there was any difficulty in providing a response, as recommended by Ferreira in 2005.^29^ None of the mothers reported such difficulties.

All the items tested in the second pretest were put into a pilot study to analyze outcomes relating to another study on child health and nutrition, which that included 50 mothers of preschool-age children from a public school in Vila Sonia and a private school in Butantã. During this phase, four trained field researchers (fourth-year nutrition students) administered the questionnaire. Within a mean interval of approximately 7 days (a minimum of 2 days and a maximum of 14 days), a re-test was conducted on 50 mothers. To evaluate the clarity of the questions, the mothers were asked to report any feelings of uncertainty by choosing one of the following responses at the end of the questionnaire: “I did not understand anything”, “I understood little”, “I understood it more or less”, “I understood almost everything, but I have some uncertainties”, or “I understood everything completely”, as recommended by Ferreira in 2005.^29^

The need to semantically adapt the items was noted. The items used in the pilot study were semantically modified by a Portuguese teacher. Thus, a final version of the questionnaire was created, which was then subjected to a third pretest with six mothers. Among these mothers, none identified any confusing elements or difficulties in responding. A total of 28 mothers participated in the pretest, which was close to Pasquali’s recommendation of 30, in 1998.^30^ None of the mothers who participated in the pretests and the pilot study participated in the final study.

The final study used the version of the questionnaire that included the changes added after the second pretest and which was tested in the third pretest. The same field researchers who collected the data in the pilot study collected the data for the final study, again by means of interviews. There was no self-administration of questionnaires. The average time taken by the researcher to fill out the questionnaire with the mother’s answers was 12 minutes (range: 5 to 19 minutes).

### Statistical analyses

The categorical variables were described according to their frequencies in percentages and 95% confidence intervals, to obtain the precision of the estimate. The mothers’ ages were described using the mean and standard deviation.

The reliability of the instrument was evaluated using two statistical measurements: for internal consistency, Cronbach’s alpha greater than 0.7 was used as the cutoff point;^31^ and for temporal stability (comparing responses in the first application with those of the re-test), a pairwise inter-observer correlation coefficient greater than 0.5 was used.^18^ The pairwise correlation coefficient assessed the agreement of the responses between the test and re-test.

Factor analysis was used to evaluate the data for patterns and reduce the many variables to a manageable number.^32^ As a prerequisite for conducting factor analysis, two tests were performed. Bartlett’s test of sphericity was used to assess the adequacy of the correlation matrix. Next, the Kaiser-Meyer-Olkin measurement of sampling adequacy was used to determine whether the correlation patterns between the variables were compact or scattered.^33^ The results from this analysis were used to determine whether the factor analysis would be capable of producing different and reliable factors (positive result ≥ 0.6).

After applying these two tests, the factor analysis was performed in three steps: identifying eigenvalues > 1; constructing a scree plot;^31^ and performing a parallel analysis. An eigenvalue greater than one indicates the amount of variance that each factor represents within the total variance.^31^^,^^32^ The second criterion, the scree plot, uses the point where the curve begins to level off as the criterion. Finally, a parallel analysis was performed, which consisted of performing 10 repetitions of the mothers’ random responses to compare the eigenvalues of the questionnaire with the eigenvalues of the parallel analysis. After the parallel analysis, oblique rotation was used to obtain the values of each item within each factor chosen (correlation of each item within each factor).^31^^,^^32^ The codes of the items with negative load values were reversed during the statistical analysis. Only those items with load values greater than 0.32 or less than -0.32 from the formula were included, as suggested by Norman and Streiner.^32^ Each factor was named using its items with the greatest load and combining the meaning of all the items that it comprised. Factor 1 was named “Personal values”, factor 2 “Family life”, factor 3 “Bohemian”, factor 4 “Socially conscious” and factor five “Modern”. Version 10.0 of the STATA statistical package was used to perform these analyses.

### Ethics committee

The research protocol was approved by the Research Ethics Committee of the Clinical Hospital of the School of Medicine at the University of São Paulo. The study was developed with the approval of the Butantã Regional Board of Education.

## RESULTS

The median age and standard deviation of the participants were 32.9 and 6.21 years, respectively. The percentages of mothers with children in public and private schools were 80% (204) and 20% (51), respectively. Table 1 shows the sample characteristics.

**Table 1.**
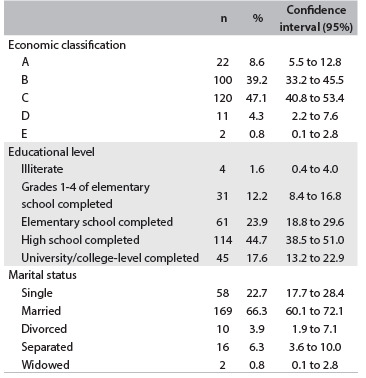
Sample characteristics according to the number, proportion and confidence interval of the economic classification, education level and marital status of the mothers of preschool-age children, southeastern region of the city of São Paulo (SP), 2011

The value of Cronbach’s alpha in the version applied in the pilot study was 0.81, and the value of the final version of the instrument (after semantic and cultural revision) was 0.83. The pairwise correlation coefficients for the test-retest also improved from the pilot study to the final application of the instrument (in each stage with 53 items), with all values greater than 0.50.

Bartlett’s test of sphericity produced a P-value < 0.001, and the Kaiser-Meyer-Olkin (KMO) measurement of sampling adequacy was 0.815. Both results confirmed that the data matrix could be subjected to factor analysis.

Factor analysis identified 53 lifestyle factors. Using both criteria (eigenvalue > 1 and scree plot), we could maintain six factors. However, the eigenvalue of the sixth factor was similar to the eigenvalue of the parallel analysis. Beyond the sixth factor, there was still a slight slope on the curve. Five factors explained 73.5% of the variance of intercorrelations. These factors were chosen to undergo oblique rotation. Table 2 shows the load values of each item within each factor, and the eigenvalues, percentage of variance and alpha coefficient of each factor.

**Table 2.**
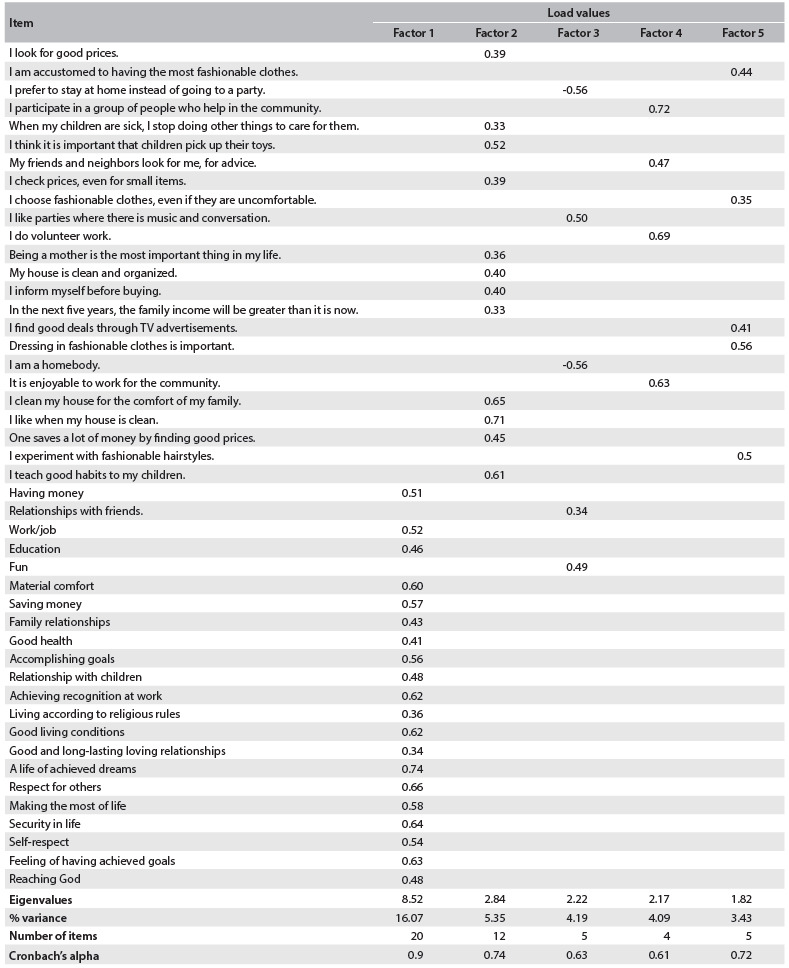
Description of the load values of each item, eigenvalue, % variance, number and alpha value of the items for each factor, southeastern region of the city of São Paulo (SP), 2011

The load values that were greater than 0.32 and less than -0.32 were selected, which resulted in elimination of seven variables from the lifestyle questionnaire. After eliminating these items, the value of Cronbach’s alpha was 0.85.

The final questionnaire with the names of the factors and each of the items with its response options is presented in Appendix A.

Factor 1 related to mothers whose actions and behaviors were based on values built throughout life. Factor 2 referred to mothers for whom the link with their homes, such as organizing the household and taking care of their children, was the most important issue. High scores in factor 3 implied that this was a person for whom leisure and pleasure were very important. Seeking fun was one of its main characteristics. Factor 4 identified mothers who were interested in helping other people.

Last but not least, factor 5 identified mothers who focused on fashion-related matters.

A mother with a bohemian lifestyle could also be a mother with a socially conscious lifestyle, i.e. none of these five factors were exclusive of the others. Rather, the factors would best be used to compare mothers with higher or lower scores for each factor.

## DISCUSSION

This study developed an easily administered instrument that characterized mothers into five lifestyles according to a sociological paradigm. It had a randomized design in which information was collected from mothers on their health and nutritional behaviors and risks. These data included information on nutritional state, diet, morbidity, physical activity and use of health services by their preschool-age children. Analyses on the correlations of these outcomes with the five lifestyles described in the present study will be presented in future articles.

The mothers’ attitudes, interests, opinions and values determined which of the five lifestyle dimensions they possessed, which then potentially influenced the health and nutrition of their preschool-age children.

Describing lifestyle is a difficult task because of the multidimensionality of its makeup and because of the inherent difficulty of measuring a subjective phenomenon in an objective manner. Previous studies in this field have used instruments for measuring healthy lifestyle behaviors;^8^ however, no studies were found that evaluated maternal lifestyle in relation to preschoolers by examining their mothers’ activities, interests, opinions and values. Marketing studies have been published that establish relationships between lifestyle dimensions and eating habits among adults.^17^

The questionnaire consisted of two sections. The first section contained 29 items relating to activities, interests and opinions. The second section contained 24 items on the mothers’ personal values.

In the pilot study, the value obtained for Cronbach’s alpha was within the acceptable range, thus demonstrating that the questionnaire presented good reliability. However, the majority of the items in the questionnaire had a pairwise correlation coefficient in the re-test that was less than 0.5. This result may have occurred because the items used adverbs and negative words in a way that made responding difficult. In addition, the items were extensive and ambiguous, and conveyed more than one idea. Bias intrinsic to questionnaire responses made it necessary to change the items in the pilot study and, following revision, the items moved in a direction that was different to what had originally been proposed. Moreover, it had to be taken into consideration that the mothers needed to remember what type of behavior they exhibited with regard to the item in question. In evaluating the item and remembering past actions, it was also necessary for mothers to estimate or infer their own behavior. Even after addressing all of these potential problems, the mothers still could have had difficulties differentiating between the types of responses.^31^

In the final study, the internal consistency assessed by Cronbach’s alpha was lower than the value of 0.92 obtained by Kucukemiroglu in 1997.^14^ In that study, the questionnaire contained 56 activity, interest and opinion items that were similar to those used in the questionnaire of the present study, and 532 respondents were tested. However, when the value is less than 0.7, the questionnaire most likely addresses a construct that is different to the one that it seeks to measure, whereas when the alpha is greater than 0.9, there is an extremely high correlation due to item redundancy.^34^^,^^35^ For three of the lifestyle factors, the value of Cronbach’s alpha was less than 0.7 in the present study. The reason for this result was the small number of items in each factor, given that the alpha can be influenced by the number of items that compose it.

The test-retest reproducibility indicated that there was stability in the respondents’ lives if the first responses were equal to or similar to those in the subsequent interview. It is important to take into consideration the time that passed between the two evaluations. In this study, the average time interval until the retest was within the recommended timeframe of 2 to 14 days.^31^^,^^32^ This timeframe was chosen to reduce the possibility of large changes and the possibility that the instrument could produce false reliability.

After conducting factor analyses to determine the existence and characteristics of the lifestyle dimensions, the phrases relating to the activity, interest, opinion and value questionnaire were subjected to varimax rotation to correct certain aspects, such as those described below. The first aspect related to the generality and the high number of items belonging to factor 1. This outcome occurred because all of the measurements were conducted on the same individuals, thus favoring correlation among the items. The second aspect involved the bipolarity of the items, which made it difficult to discern which factor an item belonged to. The third aspect was the factorial complexity of the items. In other words, without rotation, the item possessed a load value that was similar in two factors. Finally, the item might possess an average load value, whereas it would be preferable for the value to be at one of the extremes.^32^

This instrument enables measurements that can be used to compare the degree of exposure to each of the factors considered. Consequently, studies that use this instrument will be able to classify the participants into terciles of low, medium and high scores for each of the factors. This is not a questionnaire that classifies according to scores. The scoring achieved in each domain may be compared with children’s nutritional characteristics, for example. In different cultures, the exposure to a specific lifestyle may imply different outcomes. Identification of the five lifestyle dimensions will, for example, make it possible to understand how the nutritional state, eating habits, morbidity and physical activity of a child correspond to the modern or economic dimension. For example, does the fact that a mother prefers to save money cause her child to have better or worse eating habits?

The limitations of this study were that we could not proceed with criterion validation because of a lack of a reference instrument for activities, interests, opinions and values; and that the questionnaire, as it was applied, was intended for use with young adult women.

The present study is part of a larger one. A future paper will demonstrate the validation of the construct. Currently, maternal lifestyle is correlated with some behavioral patterns relating to children’s health. In this regard, maternal activities, interests, opinions and values are possible social and cultural determinants of children’s obesity and behavior disorders.

## CONCLUSIONS

The questionnaire developed here is the first instrument to describe five lifestyle domains according to the attitudes, interests, opinions and values of the mothers of preschool-age children in Brazil. The instrument content was evaluated and had good reliability as determined through Cronbach’s alpha and pairwise correlation coefficients.

The present questionnaire, “Lifestyle Activities, Interests, Opinions and Values”, is a tool that may be of great value in helping healthcare professionals to understand the motivations behind risky maternal behavior in relation to the health of preschoolers, thereby enabling interventions that may be more effective.

## References

[1] Wilson DMC, Ciliska D (1984). Lifestyle assessment: development and use of the FANTASTIC checklist. Can Fam Physician.

[2] Ciliska D, Wilson DMC (1984). Lifestyle assessment: helping patients change health behaviors. Can Fam Physician.

[3] Wilson DMC, Nielsen E, Ciliska D (1984). Lifestyle assessment: testing the FANTASTIC instrument. Can Fam Physician.

[4] Sharratt JK, Sharratt MT, Smith DM, Howell MJ, Davenport L (1984). FANTASTIC lifestyle survey of University of Waterloo employees. Can Fam Physician.

[5] Simpson R, Albert W, Wilson DMC, Ciliska D, Evans CE (1984). Lifestyle assessment: part 4. The Halton Health Promotion Survey. Can Fam Physician.

[6] Kason Y, Ylanko VJ (1984). FANTASTIC lifestyle assessment: part 5. Measuring lifestyle in family practice. Can Fam Physician.

[7] Decina PA, McGregor M, Hagino C (1990). Lifestyle analysis: a comparative study between freshman, second and fourth year chiropractic students. J Can Chiropr Assoc.

[8] Rodriguez Añez CR, Reis RS, Petroski EL (2008). Versão brasileira do questionário “estilo de vida fantástico”: tradução e validação para adultos jovens [Brazilian version of a lifestyle questionnaire: translation and validation for young adults]. Arq Bras Cardiol.

[9] Instituto Brasileiro de Geografia e Estatística (2010). Pesquisa de Orçamentos Familiares 2008-2009: Antropometria e estado nutricional de crianças, adolescentes e adultos no Brasil.

[10] Brasil. Ministério da Saúde. Centro Brasileiro de Análise e Planejamento (2009). Pesquisa Nacional de Demografia e Saúde da Criança e da Mulher PNDS 2006: Dimensões do processo reprodutivo e da saúde da criança.

[11] Goodman A, Fleitlich-Bilyk B, Patel V, Goodman R (2007). Child, family, school and community risk factors for poor mental health in Brazilian schoolchildren. J Am Acad Child Adolesc Psychiatry.

[12] World Health Organization (1998). Health promotion glossary.

[13] Kamakura WA, Wedel M (1995). Life-style segmentation with tailored interviewing. Journal of Marketing Research.

[14] Kucukemiroglu O (1999). Market segmentation by using consumer lifestyle dimensions and ethnocentrism: an empirical study. European Journal of Marketing.

[15] Wei R (1997). Emerging lifestyles in China and consequences for perception of advertising, buying behavior and consumption preferences. International Journal of Advertising.

[16] Vyncke P (2002). Lifestyle segmentation: from attitudes, interests and opinions, to values, aesthetic styles, life visions and media preferences. European Journal of Communication.

[17] Kesic T, Piri-Rajh S (2003). Market segmentation on the basis of food-related lifestyles of Croatian families. British Food Journal.

[18] Finotti MA (2009). Contribuições ao estudo dos estilos de vida: comportamento de compra e uso de crédito [thesis].

[19] Kassarjian HH (1971). Personality and consumer behavior: a review. Journal of Marketing Research.

[20] Kotler P (1998). Administração de marketing: análise, planejamento, implementação e controle.

[21] Sheth JN (2001). Comportamento do cliente: indo além do comportamento do consumidor.

[22] Divine RL, Lepisto L (2005). Analysis of the healthy lifestyle consumer. Journal of Consumer Marketing.

[23] São Paulo. Prefeitura de São Paulo. Secretaria Municipal da Saúde Estimativas Populacionais. População do município de São Paulo.

[24] Cardoso MA, Kac G, Sichieri R, Gigante DP (2007). Desenvolvimento, validação e aplicações de questionários de frequência alimentar em estudos epidemiológicos. Epidemiologia nutricional.

[25] Associação Brasileira de Estudos Populacionais (2008). Critério de classificação econômica Brasil.

[26] Reichenheim ME, Moraes CL, Kac G, Schieri R, Gigante D (2007). Desenvolvimento de instrumentos de aferição epidemiológicos. Epidemiologia nutricional.

[27] Engel JF, Blackwell RD, Miniard PW (2000). Comportamento do consumidor.

[28] Wells WD, Tigert DJ (1971). Activities, interests and opinions. Journal Advertising Research.

[29] Ferreira AF, Laurindo IM, Rodrigues PT (2008). Brazilian version of the foot health status questionnaire (FHSQ-BR): cross-cultural adaptation and evaluation of measurement properties. Clinics (Sao Paulo).

[30] Pasquali L (1998). Princípios de elaboração de escalas psicológicas [Principles of elaboration of psychological scales]. Rev Psiquiatr Clín (São Paulo).

[31] Streiner DL, Norman GR (2008). Health measurements scales: a practical guide to their development and use.

[32] Streiner DL, Norman GR (2003). Exploratory factor analysis in PDQ statistics.

[33] Fayers PM, Machin D, Staquet MJ, Hays RD, Fayers PM (1998). Factor analysis. Quality of life assessment in clinical trials. Method and practice.

[34] McDowell I (2006). Measuring health: a guide to rating scales and questionnaires.

[35] Bracher ESB (2008). Adaptação e validação da versão em português da escala graduada de dor crônica para o contexto cultural brasileiro [thesis].

